# 2-(2,7-Dimeth­oxy-1-naphtho­yl)benzoic acid

**DOI:** 10.1107/S1600536810006847

**Published:** 2010-02-27

**Authors:** Daichi Hijikata, Kosuke Nakaema, Shoji Watanabe, Akiko Okamoto, Noriyuki Yonezawa

**Affiliations:** a Department of Organic and Polymer Materials Chemistry, Tokyo University of Agriculture & Technology, Koganei, Tokyo 184-8588, Japan

## Abstract

In the title compound, C_20_H_16_O_5_, the dihedral angle between the naphthalene ring system and the benzene ring is 67.43 (5)°. The bridging carbonyl C—C(=O)—C plane makes dihedral angles of 82.64 (6) and 41.79 (7)°, respectively, with the naphthalene ring system and the benzene ring. The dihedral angle between the carb­oxy O—C(=O)—C plane and the benzene ring is 36.38 (7)° and that between the bridging carbonyl C—C(=O)—C plane and the carb­oxy O—C(=O)—C plane is 51.88 (8)°. The crystal structure is stabilized by inter­molecular O—H⋯O and C—H⋯O hydrogen-bonding inter­actions. An intra­molecular C—H⋯O hydrogen bond occurs between a naphthalene H atom and the carbonyl O atom of the carb­oxy group.

## Related literature

For electrophilic aromatic substitution of naphthalene derivatives, see: Okamoto & Yonezawa (2009 ▶). For related structures, see: Mitsui, Nakaema *et al.* (2008 ▶); Mitsui, Noguchi *et al.* (2009 ▶); Watanabe, Nakaema, Muto *et al.* (2010 ▶); Watanabe, Nakaema, Nishijima *et al.* (2010 ▶); Hijikata *et al.* (2010 ▶).
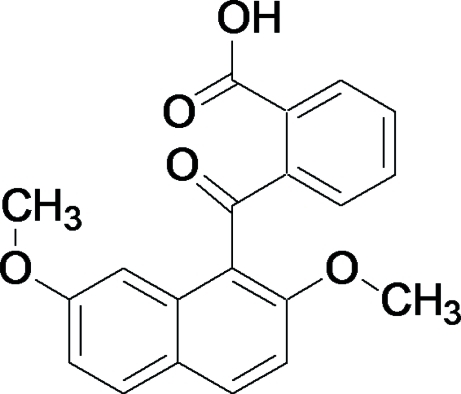


## Experimental

### 

#### Crystal data

C_20_H_16_O_5_
*M* *_r_* = 336.33
Monoclinic, 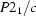

*a* = 10.8311 (2) Å
*b* = 10.61451 (19) Å
*c* = 15.4492 (3) Å
β = 111.728 (1)°
*V* = 1649.95 (5) Å^3^
*Z* = 4
Cu *K*α radiation
μ = 0.81 mm^−1^
*T* = 296 K
0.60 × 0.60 × 0.30 mm

#### Data collection

Rigaku R-AXIS- APID diffractometer
Absorption correction: multi-scan (*NUMABS*; Higashi, 1999 ▶) *T*_min_ = 0.711, *T*_max_ = 0.785
29727 measured reflections
3023 independent reflections
2710 reflections with *I* > 2σ(*I*)
*R*_int_ = 0.028

#### Refinement

*R*[*F* ^2^ > 2σ(*F* ^2^)] = 0.032
*wR*(*F* ^2^) = 0.095
*S* = 1.04
3023 reflections
233 parameters
H atoms treated by a mixture of independent and constrained refinement
Δρ_max_ = 0.16 e Å^−3^
Δρ_min_ = −0.12 e Å^−3^

### 

Data collection: *PROCESS-AUTO* (Rigaku, 1998 ▶); cell refinement: *PROCESS-AUTO*; data reduction: *CrystalStructure* (Rigaku/MSC, 2004 ▶); program(s) used to solve structure: *SIR2004* (Burla *et al.*, 2005 ▶); program(s) used to refine structure: *SHELXL97* (Sheldrick, 2008 ▶); molecular graphics: *ORTEPIII* (Burnett & Johnson, 1996 ▶); software used to prepare material for publication: *SHELXL97*.

## Supplementary Material

Crystal structure: contains datablocks global, I. DOI: 10.1107/S1600536810006847/om2323sup1.cif

Structure factors: contains datablocks I. DOI: 10.1107/S1600536810006847/om2323Isup2.hkl

Additional supplementary materials:  crystallographic information; 3D view; checkCIF report

## Figures and Tables

**Table 1:** Hydrogen-bond geometry (Å, °)

*D*—H⋯*A*	*D*—H	H⋯*A*	*D*⋯*A*	*D*—H⋯*A*
O3—H1⋯O1^i^	0.91 (2)	1.83 (2)	2.7320 (15)	173.4 (19)
C7—H7⋯O3^ii^	0.93	2.49	3.3215 (19)	150
C15—H15⋯O2^iii^	0.93	2.48	3.3152 (16)	149
C17—H17⋯O5^iv^	0.93	2.55	3.2821 (18)	136
C9—H9⋯O2	0.93	2.47	3.3850 (16)	169

Symmetry codes: (i) 
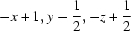
; (ii) 
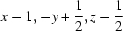
; (iii) 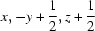; (iv) 
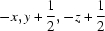
.

**Fig. 1.**
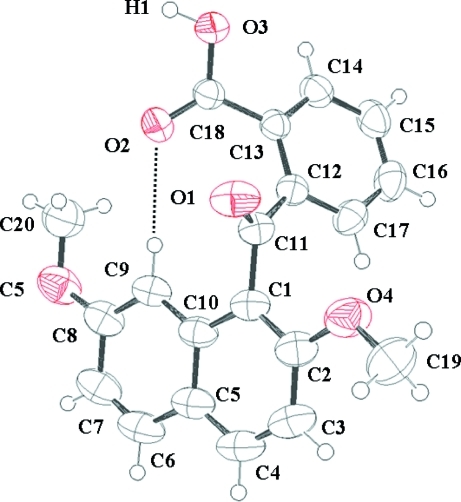
The molecular structure, showing the atom-labeling scheme and 50% probability displacement ellipsoids. The intramolecular C—H···O interaction is shown as a dashed line.

**Fig. 2.**
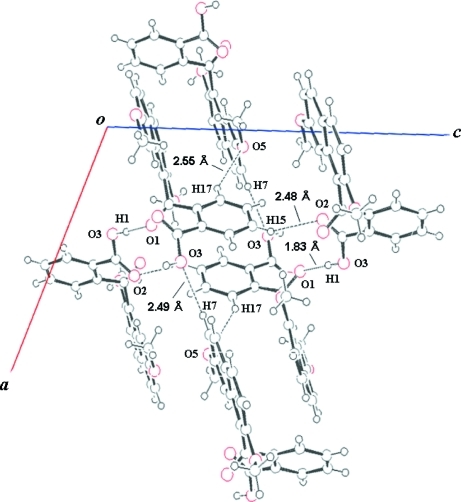
A partial crystal packing diagram viewed down b. The intermolecular O—H···O and C—H···O interactions are shown as dashed lines.

## supplementary crystallographic information

### Comment

In the course of our study on electrophilic aromatic aroylation of 2,7-dimethoxynaphthalene, *peri-*aroylnaphthalene compounds have proven to be formed regioselectively with the aid of suitable acidic mediators (Okamoto & Yonezawa, 2009). Recently, we reported the crystal structures of several 1,8-diaroylated naphthalene homologues exemplified by bis(4-bromobenzoyl)(2,7-dimethoxynaphthalene-1,8-diyl)dimethanone (Watanabe, Nakaema, Muto *et al.*, 2010). The aroyl groups at the 1,8-positions of the naphthalene rings in these compounds are twisted almost perpendicularly but the benzene ring moieties of the aroyl groups tilt slightly toward the exo sides of the naphthalene rings. Moreover, the X-ray crystal structural analysis of 1-(4-substituted benzoylated)naphthalenes, i.e., 1-(4-chlorobenzoyl)-2,7-dimethoxynaphthalene (Mitsui *et al.*, 2008), (4-chlorobenzoyl)(2-ethoxy-7-methoxynaphthalen-1-yl)methanone (Mitsui *et al.*, 2009), 1-(4-nitrobenzoyl)-2,7-dimethoxynaphthalene (Watanabe, Nakaema, Nishijima *et al.*, 2010) and methyl 4-(2,7-dimethoxy-1-naphthoyl)benzoate (Hijikata *et al.*, 2010), has also revealed essentially the same non-coplanar structure as the 1,8-diaroylated naphthalenes.

As a part of our continuing study on the molecular structures of these homologous molecules, the crystal structure of title compound, 1-monoaroylnaphthalene bearing carboxy group, is discussed in this report.

In the molecule of the title compound, shown in Fig. 1, the dihedral angle between the naphthalene ring (C1—C10) and the benzene ring (C12—C17) is 67.43 (5)°. The bridging carbonyl C1—C11(=O1)—C12 plane makes dihedral angles of 82.64 (6)° [C2—C1—C11—O1 torsion angle = -77.60 (16)°] and 41.79 (7)° [O1—C11—C12—C13 torsion angle = -37.07 (17)°], respectively, with the naphthalene ring system and the benzene ring. The dihedral angle between the carboxy O3—C18(=O2)—C13 plane and the benzene ring is 36.38 (7)° [C12—C13—C18—O2 torsion angle = -35.33 (19)°]. The dihedral angle between the bridging carbonyl C1—C11(=O1)—C12 plane and the carboxy O3—C18(=O2)—C13 plane is 51.88 (8)°. The torsion angle between one methoxy group and the naphthalene ring plane is relatively large [C29—O4—C2—C3 = -15.3 (2)°] and that between the other methoxy group and the naphthalene ring plane is rather small [C20—O5—C8—C7 = 3.8 (2)°]. The crystal structure is stabilized by intermolecular O3—H1···O1^i^ [symmetry code: (i) -x+1, y-1/2,-z+1/2] and C—H···O hydrogen-bonding interactions with one intramolecular C9—H9···O2 hydrogen bonding (Table 1, Fig 1, 2).

### Experimental

The title compound was prepared by AlCl_3_-mediated regioselective electrophilic aromatic aroylation of 2,7-dimethoxynaphthalene with acetic anhydride. Single crystals suitable for X-ray diffraction were obtained by recrystallization from diethyl ether.

Spectroscopic Data: ^1^H NMR (300 MHz, CDCl_3_) *δ*3.65 (3*H*, s), 3.78 (3*H*, s), 7.00 (1*H*, dd, *J* = 2.4, 9.0 Hz), 7.43-7.58 (4*H*, m), 7.66 (1*H*, d, *J* = 9.0 Hz), 7.84-7.88 (2*H*, m); ^13^C NMR (75.0 MHz, CDCl_3_) *δ*55.3, 56.5, 102.7, 110.5, 117.4, 124.4, 129.4, 129.5, 129.9, 130.9, 131.4, 133.8, 142.1, 157.1, 159.6, 198.0; IR (KBr): 1698, 1627, 1512; HRMS (*m*/*z*): [M+H]^+^ Calcd for C_20_H_17_O_5,_ 337.1076; Found, 337.1057.

### Refinement

All H atoms were found in a difference map and were subsequently refined as riding atoms, with C—H = 0.93 (aromatic) and 0.96 (methyl) Å, and with *U*_ĩso_(H) = 1.2*U*_eq_(C).

### Crystal data

**Table:**

C_20_H_16_O_5_	*F*(000) = 704
*M**_r_* = 336.33	*D*_x_ = 1.354 Mg m^−^^3^
Monoclinic, *P*2_1_/*c*	Cu *K*α radiation, λ = 1.54187 Å
Hall symbol: -P 2ybc	Cell parameters from 26822 reflections
*a* = 10.8311 (2) Å	θ = 3.1–68.2°
*b* = 10.61451 (19) Å	µ = 0.81 mm^−^^1^
*c* = 15.4492 (3) Å	*T* = 296 K
β = 111.728 (1)°	Block, colorless
*V* = 1649.95 (5) Å^3^	0.60 × 0.60 × 0.30 mm
*Z* = 4	

### Data collection

**Table:**

Rigaku R-AXIS- APID diffractometer	3023 independent reflections
Radiation source: rotating anode	2710 reflections with *I* > 2σ(*I*)
graphite	*R*_int_ = 0.028
Detector resolution: 10.00 pixels mm^-1^	θ_max_ = 68.2°, θ_min_ = 4.4°
ω scans	*h* = −13→13
Absorption correction: multi-scan (*NUMABS*; Higashi, 1999)	*k* = −12→12
*T*_min_ = 0.711, *T*_max_ = 0.785	*l* = −18→18
29727 measured reflections	

### Refinement

**Table:**

Refinement on *F*^2^	Secondary atom site location: difference Fourier map
Least-squares matrix: full	Hydrogen site location: difference Fourier map
*R*[*F*^2^ > 2σ(*F*^2^)] = 0.032	H atoms treated by a mixture of independent and constrained refinement
*wR*(*F*^2^) = 0.095	*w* = 1/[σ^2^(*F*_o_^2^) + (0.0481*P*)^2^ + 0.2862*P*] where *P* = (*F*_o_^2^ + 2*F*_c_^2^)/3
*S* = 1.04	(Δ/σ)_max_ < 0.001
3023 reflections	Δρ_max_ = 0.16 e Å^−^^3^
233 parameters	Δρ_min_ = −0.11 e Å^−^^3^
0 restraints	Extinction correction: *SHELXL97* (Sheldrick, 2008), Fc^*^=kFc[1+0.001xFc^2^λ^3^/sin(2θ)]^-1/4^
Primary atom site location: structure-invariant direct methods	Extinction coefficient: 0.0021 (3)

### Special details

**Table:**

Geometry. All esds (except the esd in the dihedral angle between two l.s. planes) are estimated using the full covariance matrix. The cell esds are taken into account individually in the estimation of esds in distances, angles and torsion angles; correlations between esds in cell parameters are only used when they are defined by crystal symmetry. An approximate (isotropic) treatment of cell esds is used for estimating esds involving l.s. planes.
Refinement. Refinement of *F*^2^ against ALL reflections. The weighted *R*-factor wR and goodness of fit *S* are based on *F*^2^, conventional *R*-factors *R* are based on *F*, with *F* set to zero for negative *F*^2^. The threshold expression of *F*^2^ > σ(*F*^2^) is used only for calculating *R*-factors(gt) etc. and is not relevant to the choice of reflections for refinement. *R*-factors based on *F*^2^ are statistically about twice as large as those based on *F*, and *R*- factors based on ALL data will be even larger.

### Fractional atomic coordinates and isotropic or equivalent isotropic displacement parameters (Å^2^)

**Table:**

	*x*	*y*	*z*	*U*_iso_*/*U*_eq_	
O1	0.40529 (9)	0.47494 (10)	0.24166 (7)	0.0630 (3)	
O2	0.36166 (9)	0.19907 (10)	0.24736 (6)	0.0647 (3)	
O3	0.54599 (9)	0.15634 (11)	0.36707 (7)	0.0709 (3)	
H1	0.5589 (19)	0.100 (2)	0.3272 (14)	0.104 (6)*	
O4	0.30544 (10)	0.73160 (9)	0.28563 (8)	0.0726 (3)	
O5	−0.05580 (10)	0.17144 (10)	0.06622 (8)	0.0725 (3)	
C1	0.18889 (11)	0.54939 (12)	0.21895 (8)	0.0487 (3)	
C2	0.18877 (13)	0.67861 (13)	0.22803 (10)	0.0580 (3)	
C3	0.07232 (16)	0.74830 (15)	0.18184 (12)	0.0706 (4)	
H3	0.0716	0.8349	0.1906	0.085*	
C4	−0.03897 (15)	0.68890 (15)	0.12452 (11)	0.0707 (4)	
H4	−0.1149	0.7362	0.0937	0.085*	
C5	−0.04262 (13)	0.55796 (14)	0.11051 (9)	0.0590 (3)	
C6	−0.15566 (13)	0.49476 (17)	0.04818 (11)	0.0711 (4)	
H6	−0.2311	0.5412	0.0148	0.085*	
C7	−0.15665 (14)	0.36879 (17)	0.03601 (11)	0.0710 (4)	
H7	−0.2324	0.3292	−0.0048	0.085*	
C8	−0.04226 (12)	0.29720 (14)	0.08549 (9)	0.0576 (3)	
C9	0.06985 (11)	0.35343 (12)	0.14630 (9)	0.0505 (3)	
H9	0.1442	0.3050	0.1787	0.061*	
C10	0.07285 (11)	0.48552 (12)	0.16004 (8)	0.0495 (3)	
C11	0.31457 (11)	0.47747 (11)	0.27012 (8)	0.0447 (3)	
C12	0.33101 (10)	0.41688 (11)	0.36061 (7)	0.0409 (3)	
C13	0.40131 (10)	0.30406 (11)	0.39141 (7)	0.0402 (3)	
C14	0.43259 (12)	0.26655 (13)	0.48298 (8)	0.0511 (3)	
H14	0.4795	0.1921	0.5037	0.061*	
C15	0.39508 (14)	0.33816 (15)	0.54409 (9)	0.0605 (4)	
H15	0.4171	0.3121	0.6055	0.073*	
C16	0.32540 (13)	0.44760 (14)	0.51365 (9)	0.0607 (4)	
H16	0.3004	0.4960	0.5546	0.073*	
C17	0.29207 (12)	0.48650 (12)	0.42223 (9)	0.0523 (3)	
H17	0.2431	0.5600	0.4019	0.063*	
C18	0.43300 (11)	0.21726 (11)	0.32640 (7)	0.0424 (3)	
C19	0.32145 (18)	0.86387 (15)	0.28282 (13)	0.0792 (5)	
H19A	0.4114	0.8861	0.3201	0.095*	
H19B	0.2620	0.9052	0.3069	0.095*	
H19C	0.3017	0.8900	0.2196	0.095*	
C20	0.05113 (17)	0.09279 (16)	0.11674 (13)	0.0798 (5)	
H20A	0.0300	0.0072	0.0966	0.096*	
H20B	0.0671	0.0990	0.1820	0.096*	
H20C	0.1293	0.1185	0.1061	0.096*	

### Atomic displacement parameters (Å^2^)

**Table:**

	*U*^11^	*U*^22^	*U*^33^	*U*^12^	*U*^13^	*U*^23^
O1	0.0532 (5)	0.0731 (6)	0.0744 (6)	0.0134 (4)	0.0372 (5)	0.0261 (5)
O2	0.0577 (5)	0.0842 (7)	0.0423 (5)	0.0184 (5)	0.0068 (4)	−0.0127 (4)
O3	0.0549 (5)	0.0910 (8)	0.0539 (5)	0.0303 (5)	0.0051 (4)	−0.0173 (5)
O4	0.0693 (6)	0.0470 (5)	0.0925 (8)	−0.0002 (5)	0.0194 (6)	0.0074 (5)
O5	0.0589 (6)	0.0676 (6)	0.0757 (7)	−0.0136 (5)	0.0073 (5)	0.0077 (5)
C1	0.0445 (6)	0.0497 (7)	0.0544 (7)	0.0074 (5)	0.0212 (5)	0.0128 (5)
C2	0.0566 (8)	0.0524 (7)	0.0659 (8)	0.0054 (6)	0.0238 (6)	0.0107 (6)
C3	0.0750 (10)	0.0527 (8)	0.0863 (10)	0.0201 (7)	0.0326 (8)	0.0185 (7)
C4	0.0591 (8)	0.0723 (10)	0.0796 (10)	0.0264 (7)	0.0245 (8)	0.0246 (8)
C5	0.0472 (7)	0.0696 (9)	0.0616 (8)	0.0142 (6)	0.0216 (6)	0.0198 (6)
C6	0.0434 (7)	0.0907 (12)	0.0713 (9)	0.0153 (7)	0.0121 (6)	0.0229 (8)
C7	0.0430 (7)	0.0918 (12)	0.0670 (9)	−0.0025 (7)	0.0073 (6)	0.0146 (8)
C8	0.0459 (7)	0.0673 (8)	0.0574 (7)	−0.0049 (6)	0.0165 (6)	0.0115 (6)
C9	0.0398 (6)	0.0567 (7)	0.0531 (7)	0.0037 (5)	0.0151 (5)	0.0141 (5)
C10	0.0411 (6)	0.0580 (7)	0.0518 (6)	0.0067 (5)	0.0200 (5)	0.0138 (5)
C11	0.0402 (6)	0.0435 (6)	0.0520 (6)	−0.0009 (5)	0.0190 (5)	0.0036 (5)
C12	0.0340 (5)	0.0447 (6)	0.0449 (6)	−0.0026 (4)	0.0154 (4)	−0.0013 (5)
C13	0.0345 (5)	0.0464 (6)	0.0388 (5)	−0.0007 (4)	0.0125 (4)	−0.0013 (4)
C14	0.0502 (7)	0.0599 (7)	0.0410 (6)	0.0052 (6)	0.0143 (5)	0.0036 (5)
C15	0.0610 (8)	0.0820 (10)	0.0397 (6)	0.0000 (7)	0.0200 (6)	−0.0030 (6)
C16	0.0622 (8)	0.0724 (9)	0.0560 (7)	−0.0029 (7)	0.0319 (6)	−0.0168 (6)
C17	0.0493 (7)	0.0511 (7)	0.0623 (7)	0.0009 (5)	0.0274 (6)	−0.0056 (6)
C18	0.0383 (5)	0.0476 (6)	0.0398 (6)	0.0030 (5)	0.0128 (5)	0.0003 (5)
C19	0.0928 (12)	0.0515 (8)	0.0992 (12)	−0.0041 (8)	0.0423 (10)	0.0056 (8)
C20	0.0753 (10)	0.0634 (9)	0.0870 (11)	−0.0022 (8)	0.0142 (8)	−0.0008 (8)

### Geometric parameters (Å, °)

**Table:**

O1—C11	1.2163 (13)	C8—C9	1.3670 (18)
O2—C18	1.1945 (13)	C9—C10	1.4166 (18)
O3—C18	1.3195 (14)	C9—H9	0.9300
O3—H1	0.91 (2)	C11—C12	1.4885 (16)
O4—C2	1.3684 (17)	C12—C17	1.3884 (16)
O4—C19	1.4173 (18)	C12—C13	1.4042 (16)
O5—C8	1.3637 (18)	C13—C14	1.3856 (16)
O5—C20	1.4065 (19)	C13—C18	1.4935 (15)
C1—C2	1.3788 (19)	C14—C15	1.3849 (18)
C1—C10	1.4215 (17)	C14—H14	0.9300
C1—C11	1.5046 (15)	C15—C16	1.370 (2)
C2—C3	1.4074 (19)	C15—H15	0.9300
C3—C4	1.358 (2)	C16—C17	1.3850 (19)
C3—H3	0.9300	C16—H16	0.9300
C4—C5	1.405 (2)	C17—H17	0.9300
C4—H4	0.9300	C19—H19A	0.9600
C5—C6	1.415 (2)	C19—H19B	0.9600
C5—C10	1.4256 (16)	C19—H19C	0.9600
C6—C7	1.350 (2)	C20—H20A	0.9600
C6—H6	0.9300	C20—H20B	0.9600
C7—C8	1.4126 (19)	C20—H20C	0.9600
C7—H7	0.9300		
			
C18—O3—H1	110.5 (12)	C12—C11—C1	118.89 (9)
C2—O4—C19	118.76 (12)	C17—C12—C13	119.07 (10)
C8—O5—C20	117.39 (11)	C17—C12—C11	116.93 (11)
C2—C1—C10	120.37 (11)	C13—C12—C11	123.23 (10)
C2—C1—C11	119.08 (11)	C14—C13—C12	119.21 (10)
C10—C1—C11	120.53 (11)	C14—C13—C18	118.63 (10)
O4—C2—C1	116.08 (12)	C12—C13—C18	121.94 (9)
O4—C2—C3	123.52 (13)	C15—C14—C13	121.04 (12)
C1—C2—C3	120.37 (14)	C15—C14—H14	119.5
C4—C3—C2	119.94 (14)	C13—C14—H14	119.5
C4—C3—H3	120.0	C16—C15—C14	119.65 (12)
C2—C3—H3	120.0	C16—C15—H15	120.2
C3—C4—C5	121.82 (13)	C14—C15—H15	120.2
C3—C4—H4	119.1	C15—C16—C17	120.35 (12)
C5—C4—H4	119.1	C15—C16—H16	119.8
C4—C5—C6	122.79 (13)	C17—C16—H16	119.8
C4—C5—C10	118.80 (13)	C16—C17—C12	120.65 (12)
C6—C5—C10	118.40 (13)	C16—C17—H17	119.7
C7—C6—C5	121.64 (13)	C12—C17—H17	119.7
C7—C6—H6	119.2	O2—C18—O3	122.85 (11)
C5—C6—H6	119.2	O2—C18—C13	124.36 (10)
C6—C7—C8	119.83 (14)	O3—C18—C13	112.69 (9)
C6—C7—H7	120.1	O4—C19—H19A	109.5
C8—C7—H7	120.1	O4—C19—H19B	109.5
O5—C8—C9	125.01 (12)	H19A—C19—H19B	109.5
O5—C8—C7	113.99 (13)	O4—C19—H19C	109.5
C9—C8—C7	121.00 (14)	H19A—C19—H19C	109.5
C8—C9—C10	120.03 (11)	H19B—C19—H19C	109.5
C8—C9—H9	120.0	O5—C20—H20A	109.5
C10—C9—H9	120.0	O5—C20—H20B	109.5
C9—C10—C1	122.26 (10)	H20A—C20—H20B	109.5
C9—C10—C5	119.11 (12)	O5—C20—H20C	109.5
C1—C10—C5	118.59 (12)	H20A—C20—H20C	109.5
O1—C11—C12	119.88 (10)	H20B—C20—H20C	109.5
O1—C11—C1	121.07 (10)		
			
C19—O4—C2—C1	166.64 (13)	C6—C5—C10—C9	−0.91 (18)
C19—O4—C2—C3	−15.3 (2)	C4—C5—C10—C1	−2.41 (18)
C10—C1—C2—O4	−179.44 (11)	C6—C5—C10—C1	176.71 (12)
C11—C1—C2—O4	−1.13 (18)	C2—C1—C11—O1	−77.60 (16)
C10—C1—C2—C3	2.4 (2)	C10—C1—C11—O1	100.70 (14)
C11—C1—C2—C3	−179.30 (12)	C2—C1—C11—C12	97.80 (14)
O4—C2—C3—C4	178.89 (14)	C10—C1—C11—C12	−83.90 (14)
C1—C2—C3—C4	−3.1 (2)	O1—C11—C12—C17	132.79 (12)
C2—C3—C4—C5	1.0 (2)	C1—C11—C12—C17	−42.67 (15)
C3—C4—C5—C6	−177.30 (14)	O1—C11—C12—C13	−37.07 (17)
C3—C4—C5—C10	1.8 (2)	C1—C11—C12—C13	147.48 (11)
C4—C5—C6—C7	−179.97 (15)	C17—C12—C13—C14	−1.43 (16)
C10—C5—C6—C7	0.9 (2)	C11—C12—C13—C14	168.22 (11)
C5—C6—C7—C8	−0.7 (2)	C17—C12—C13—C18	173.01 (10)
C20—O5—C8—C9	3.8 (2)	C11—C12—C13—C18	−17.34 (16)
C20—O5—C8—C7	−176.41 (14)	C12—C13—C14—C15	0.31 (18)
C6—C7—C8—O5	−179.36 (14)	C18—C13—C14—C15	−174.32 (11)
C6—C7—C8—C9	0.4 (2)	C13—C14—C15—C16	0.3 (2)
O5—C8—C9—C10	179.34 (12)	C14—C15—C16—C17	0.2 (2)
C7—C8—C9—C10	−0.4 (2)	C15—C16—C17—C12	−1.4 (2)
C8—C9—C10—C1	−176.87 (11)	C13—C12—C17—C16	1.96 (17)
C8—C9—C10—C5	0.67 (18)	C11—C12—C17—C16	−168.33 (11)
C2—C1—C10—C9	177.90 (12)	C14—C13—C18—O2	139.14 (13)
C11—C1—C10—C9	−0.38 (17)	C12—C13—C18—O2	−35.33 (18)
C2—C1—C10—C5	0.35 (17)	C14—C13—C18—O3	−37.23 (15)
C11—C1—C10—C5	−177.93 (11)	C12—C13—C18—O3	148.30 (11)
C4—C5—C10—C9	179.96 (12)		

### Hydrogen-bond geometry (Å, °)

**Table:**

*D*—H···*A*	*D*—H	H···*A*	*D*···*A*	*D*—H···*A*
O3—H1···O1^i^	0.91 (2)	1.83 (2)	2.7320 (15)	173.4 (19)
C7—H7···O3^ii^	0.93	2.49	3.3215 (19)	150
C15—H15···O2^iii^	0.93	2.48	3.3152 (16)	149
C17—H17···O5^iv^	0.93	2.55	3.2821 (18)	136
C9—H9···O2	0.93	2.47	3.3850 (16)	169

Symmetry codes: (i) −*x*+1, *y*−1/2, −*z*+1/2; (ii) *x*−1, −*y*+1/2, *z*−1/2; (iii) *x*, −*y*+1/2, *z*+1/2; (iv) −*x*, *y*+1/2, −*z*+1/2.
